# Soil Bacterial Community Structure and Functional Potential in the Caspian Drylands of Western Kazakhstan

**DOI:** 10.3390/biology15120969

**Published:** 2026-06-20

**Authors:** Yryszhan Zhakypbek, Murat Toktar, Bekzhan D. Kossalbayev, Qiuli Yang, Qingdong Shi, Serik Tursbekov, Ayaz M. Belkozhayev, Altynbek S. Abseyt, Gulmira Kezembayeva, Tileu Kamarkhan

**Affiliations:** 1 Department of Mine Surveying and Geodesy, Institute Mining and Metallurgical Institute Named After O.A. Baikonurov, Satbayev University, Almaty 050013, Kazakhstan; y.zhakypbek@satbayev.university (Y.Z.); murat-toktar@mail.ru (M.T.); s.tursbekov@satbayev.university (S.T.); g.kezembayeva@satbayev.university (G.K.); 2 Department of Chemical and Biochemical Engineering, Geology and Oil-Gas Business Institute Named After K. Turyssov, Satbayev University, Almaty 050043, Kazakhstan; 3 Ecology Research Institute, Khoja Akhmet Yassawi International Kazakh Turkish University, Turkistan 161200, Kazakhstan; altynbek.abseyt@bk.ru; 4 College of Geography and Remote Sensing Science, Xinjiang University, Urumqi 830049, China; yangqiuli@xju.edu.cn; 5 Xinjiang Key Laboratory of Oasis Ecology, Xinjiang University, Urumqi 830046, China; shiqd@xju.edu.cn; 6 Xinjiang Field Scientific Observation and Research Station for the Oasisization Process in the Hinterland of the Taklamakan Desert, Yutian 848400, China; 7 College of Ecology and Environment, Xinjiang University, Urumqi 830046, China; 8 Almaty Technical and Economic College of Transport Communications, Almaty 050019, Kazakhstan; kamarkantileu@gmail.com

**Keywords:** bacterial community, 16S rRNA amplicon sequencing, alkaline dryland soil, *Rubrobacter*, *Marinobacter*, FAPROTAX, predicted functional potential, microbial community composition

## Abstract

Dryland soils of the Caspian region of western Kazakhstan are exposed to environmental stress, including drought, alkalinity, low soil organic matter content, and anthropogenic pressure. In this preliminary study, bacterial communities were investigated in 18 soil samples collected from six sampling groups across Makat (M1, M2), Isatay (I1, I2), and Beyneu (B1, B2) districts. Soil physicochemical properties were measured, and bacterial diversity was analyzed using 16S rRNA gene sequencing of the V3–V4 region. Community composition analysis indicated spatial heterogeneity among the sampled groups. M1 and I1 showed the highest taxon richness, whereas B2 contained the highest number of unique taxa. Genus-level profiles showed that B1 and M2 were mainly associated with *Rubrobacter* and related actinobacterial taxa; B2 contained higher proportions of *Marinobacter*, *Tychonema*, *Qipengyuania*, and *Halomonas*; and I2 was enriched with *Antarcticibacterium*, *Salinimicrobium*, *Rhodococcus*, *Gillisia*, *Marinobacter*, *Dietzia*, and *Pontibacter*. Correlation analysis showed that several bacterial taxa were associated with soil organic matter content, total nitrogen, total phosphorus, exchangeable cations, and pH, although the overall Mantel relationship between soil properties and community structure was not significant. FAPROTAX-based prediction indicated differences in putative heterotrophic, nitrogen-related, sulfur-related, and hydrocarbon-associated functional categories among sites. Because FAPROTAX predictions are based on taxonomic composition, these results should be interpreted only as putative functional potential and not as evidence of actual microbial metabolic activity. These findings suggest that the sampled Caspian dryland soils contain distinct bacterial assemblages and taxa with potential ecological relevance; however, their role in dryland soil resilience or bioremediation should be verified through future culture-based, metagenomic, and functional validation studies.

## Simple Summary

Dryland soils in the Caspian region of western Kazakhstan face drought, high alkalinity, low organic matter, and pressure from human activity, but little is known about the bacteria that live in these soils and help maintain soil health. This study examined soil from Makat, Isatay, and Beyneu to understand which bacteria are present, how they differ among locations, and how they may relate to basic soil properties such as acidity or alkalinity, nutrients, and mineral content. The results showed that the soils were alkaline and that bacterial communities differed clearly among sampling sites. Some sites had higher bacterial richness, while others contained more unique bacterial groups. Several bacteria were linked with soil nutrients and minerals, and some belonged to groups that may be useful in future studies of dryland soil recovery or pollution cleanup. However, the study did not directly measure salt, oil pollution, or bacterial activity, so these possibilities need further testing. In addition, because the study was based on one-time sampling, the results do not reflect seasonal dynamics of soil bacterial communities.

## 1. Introduction

Dryland soils are among the most vulnerable terrestrial ecosystems because they are exposed to low precipitation, high evaporation, strong temperature fluctuations, low soil carbon and soil organic matter reserves, salt accumulation risk, and frequent nutrient limitation [1]. Under these conditions, soil biological processes depend strongly on microorganisms, especially bacteria. Soil bacteria contribute to residue decomposition, carbon transformation, nitrogen and phosphorus cycling, mineral weathering, soil aggregation, and plant productivity. Although soil bacterial communities are highly diverse, their distribution is not random; rather, bacterial assemblages are filtered by climate, soil chemistry, water availability, vegetation, and disturbance history [2,3]. Therefore, studying bacterial communities in dry regions is essential for understanding land degradation, ecosystem stability, and the biological potential of stressed soils.

Bacterial communities in arid and semi-arid environments are particularly important because they include taxa capable of persisting under water deficit, alkaline pH, ultraviolet radiation, nutrient scarcity, and osmotic stress. Recent studies show that dryland microbiomes provide key ecosystem functions but are increasingly threatened by desertification and climate change [4]. Global analyses of arid soils have reported recurrent enrichment of Actinobacteria and other stress-tolerant bacterial groups, with community patterns often related to temperature, precipitation, soil chemistry, and habitat structure [5,6]. Neilson et al. showed that increasing aridity can alter bacterial diversity, phylogenetic composition, and microbial connectivity, potentially affecting nutrient cycling and soil functional stability [7]. Similarly, studies of desert–oasis gradients have shown that bacterial diversity and community composition can vary strongly across short spatial distances, reflecting differences in soil moisture, vegetation, and nutrient availability [8,9].

The Kazakhstan Caspian region is a relevant area for dryland microbial ecology because it combines natural aridity, alkaline soils, land degradation risk, and long-term anthropogenic pressure. Previous studies have reported environmental concerns in the wider Caspian region, including soil salinization, desertification, water-quality decline, heavy metal accumulation, and petroleum-related disturbance. However, these issues are presented here only as regional environmental context. The present study did not directly measure petroleum hydrocarbons, PAHs, trace metals, electrical conductivity, soluble salts, chloride, or sulfate, and therefore it was not designed to assess oil contamination or salinity effects. Instead, the study aimed to characterize bacterial community composition and putative functional potential in dryland soils from Makat, Isatay, and Beyneu and to evaluate their associations with the measured soil physicochemical properties. Previous studies reported pollutants such as heavy metals, oil contaminants, pesticides, and polychlorinated biphenyls in the Ural–Caspian basin [10]. Other work showed that the northern Caspian Sea area bounded by Kazakhstan is affected by water-quality risks, including cadmium contamination [11]. Studies of oil-contaminated soils in Kazakhstan have also shown that petroleum contamination can affect bacterial communities and that indigenous microorganisms may include taxa with hydrocarbon-degradation potential [12].

Despite this regional importance, information about bacterial communities in dryland soils of the Kazakhstan Caspian region remains limited. Many previous studies focused on water contamination, oil degradation, or individual polluted sites, whereas fewer studies have combined soil physicochemical characterization with 16S rRNA gene sequencing across multiple dryland locations. In particular, there is still limited information on how bacterial richness, dominant taxa, site-specific taxa, and predicted functional profiles vary among dryland soils from Makat, Isatay, and Beyneu. Such information is needed as a baseline for future monitoring, especially because bacterial taxa detected by amplicon sequencing may help identify candidate groups for subsequent culture-based, metagenomic, and contaminant-focused studies. Because detailed measurements of vegetation structure, soil moisture, electrical conductivity, soluble salts, and contaminants were not included, these ecological differences are used only as contextual information and not as direct explanatory variables [13].

The aim of this study was to characterize bacterial community composition and putative functional potential in dryland soils from the Kazakhstan Caspian region and to evaluate their associations with measured soil physicochemical properties. We analyzed 18 soil samples from six sampling groups across Makat, Isatay, and Beyneu, measured pH, soil organic matter content, total nitrogen, total phosphorus, available phosphorus, available potassium, and exchangeable Ca, Mg, and Na, and assessed bacterial communities using 16S rRNA gene sequencing of the V3–V4 region. Because petroleum hydrocarbons, PAHs, trace metals, electrical conductivity, and soluble salts were not measured, any discussion of oil-, salinity-, or hydrocarbon-associated taxa is treated only as exploratory and hypothesis-generating.

We hypothesized that bacterial community composition would differ among the six sampling groups and that these differences would be associated with measured soil physicochemical properties, especially pH, soil organic matter content, nutrient status, and exchangeable cations. We further expected that alkaline and nutrient-poor soils would contain higher proportions of taxa commonly reported from dryland or stress-prone environments, whereas some sites might contain taxa that include species previously reported from saline, mineralized, or hydrocarbon-affected habitats. Because electrical conductivity, soluble salts, chloride, sulfate, petroleum hydrocarbons, PAHs, and trace metals were not measured in this study, salinity- and hydrocarbon-related interpretations are treated as exploratory and hypothesis-generating rather than as confirmed environmental effects.

## 2. Materials and Methods

### 2.1. Study Area and Soil Sampling

Soil samples were collected from three dryland districts of western Kazakhstan: Isatay District, Atyrau Region; Makat District, Atyrau Region; and Beyneu District, Mangystau Region. According to the sampling map, the study areas were separated by approximately 180 km between Isatay and Makat, 295 km between Makat and Beyneu, and 385 km between Isatay and Beyneu. Six sampling groups were investigated: M1, M2, B1, B2, I1, and I2. To improve metadata transparency, detailed information for each sampling group, including coordinates, land use, vegetation cover, soil type, visible disturbance, and distance to the nearest industrial or oilfield source, is provided in Supplementary Table S1. Sampling points were selected to represent the dominant dryland soil conditions of each area and to avoid visibly disturbed microsites such as roadsides, waste sites, animal burrows, or recently excavated soil. Environmental metadata were organized following the logic of MIxS/MIMARKS-style reporting for marker-gene studies [14,15].

A biological replicate was defined as one independent field sampling point within a sampling group. Three biological replicates were collected from each sampling group, resulting in 18 biological replicates in total. At each biological replicate point, soil was collected from two depth intervals: 0–10 cm and 10–20 cm. Equal masses of soil from the two depth intervals were homogenized to prepare one composite 0–20 cm sample for each biological replicate. Therefore, the final dataset consisted of 18 composite samples [16,17,18].

Approximately 200 g of soil was collected from each depth interval using sterile stainless-steel tools. After collection, samples were placed in sterile polyethylene bags, transported to the laboratory in clean containers, and processed immediately. Visible roots, stones, and plant residues were removed manually. Samples were homogenized and passed through a 2 mm sieve. Subsamples used for physicochemical analyses were air-dried at room temperature and further ground to pass a 0.25 mm sieve where required. Subsamples used for DNA extraction were homogenized separately and stored under clean conditions until molecular analysis [19,20].

All physicochemical measurements were performed on a dry-soil basis. Analytical measurements were performed in at least three technical repetitions per biological replicate.

### 2.2. Soil Physicochemical Analyses

Soil pH was measured potentiometrically in a 1:2.5 soil-to-water suspension. Briefly, 10 g of air-dried soil was mixed with 25 mL of deionized water, shaken for 30 min, allowed to equilibrate, and measured using a calibrated glass-electrode pH meter. The pH meter was calibrated before measurement using standard buffer solutions at pH 4.01, 7.00, and 10.01.

Soil organic matter was determined by the Tyurin dichromate oxidation method. In brief, air-dried and finely ground soil was oxidized with potassium dichromate in concentrated sulfuric acid, and the remaining dichromate was back-titrated with ferrous ammonium sulfate. Oxidizable organic carbon was converted to soil organic matter using the Van Bemmelen factor of 1.724.

Total nitrogen was determined using the Kjeldahl digestion–distillation–titration method. Soil was digested with concentrated sulfuric acid in the presence of a catalyst mixture, after which ammonium nitrogen was distilled under alkaline conditions, trapped in boric acid solution, and titrated with standardized hydrochloric acid [21].

Total phosphorus was measured after acid digestion followed by molybdenum blue spectrophotometry. Soil samples were digested using a nitric–perchloric acid mixture until a clear digest was obtained. Phosphorus in the digest was determined colorimetrically using the molybdenum blue reaction and measured with a UV–Vis spectrophotometer (UV-2600i Plus, Shimadzu Scientific Instruments, Columbia, MD, USA).

Available phosphorus was determined using the Machigin method for carbonate and calcareous soils. Soil phosphorus was extracted with 1% ammonium carbonate solution, and phosphorus in the extract was quantified by the molybdenum blue spectrophotometric method [22,23].

Exchangeable Ca, Mg, Na, and available/exchangeable K were extracted using 1 M ammonium acetate buffered at pH 7.0. Soil was extracted at a 1:10 soil-to-solution ratio by shaking for 1 h, followed by filtration. Potassium and sodium were quantified by flame photometry, whereas calcium and magnesium were determined by atomic absorption spectrometry. Calibration curves were prepared using certified standard solutions for each element, and blanks were included for quality control [24,25,26,27,28].

Electrical conductivity, total soluble salts, carbonate content, particle-size distribution, gravimetric moisture, chloride, and sulfate were not measured in the original experimental design.

Each basic soil chemical parameter was determined in three technical repetitions for each biological replicate, and the final values were used to calculate group means and standard deviations.

### 2.3. DNA Extraction, Amplification, and Sequencing

Genomic DNA was extracted from 0.5 g of homogenized soil using the E.Z.N.A.^®^ Soil DNA Kit (Omega Bio-tek, Norcross, GA, USA), following the manufacturer’s protocol. DNA concentration and purity were assessed using a NanoDrop 2000 spectrophotometer (Thermo Fisher Scientific, Wilmington, DE, USA) and Qubit dsDNA HS Assay Kit (Life Technologies Corporation, Eugene, OR, USA), and DNA integrity was checked by 1% agarose gel electrophoresis. Extraction blanks and no-template PCR controls were processed in parallel to monitor potential background contamination [29,30].

The V3–V4 region of the bacterial 16S rRNA gene was amplified using primers 338F, 5′-ACTCCTACGGGAGGCAGCAG-3′, and 806R, 5′-GGACTACHVGGGTWTCTAAT-3′. PCR amplification was performed in 20 μL reactions containing 4 μL of 5× FastPfu buffer, 2 μL of 2.5 mM dNTPs, 0.8 μL of each primer, 0.4 μL of FastPfu DNA polymerase, approximately 10 ng of template DNA, and nuclease-free water. The PCR cycling program consisted of initial denaturation at 95 °C for 3 min; 27 cycles of denaturation at 95 °C for 30 s, annealing at 55 °C for 30 s, and extension at 72 °C for 45 s; followed by a final extension at 72 °C for 10 min.

PCR products were checked by 2% agarose gel electrophoresis. Amplicons of the expected size were purified using the AxyPrep DNA Gel Extraction Kit (Axygen Biosciences, Union City, CA, USA), quantified using the QuantiFluor™-ST fluorometric system (Promega Corporation, Madison, WI, USA), and pooled in equimolar concentrations. Sequencing libraries were prepared using a dual-index barcode strategy and sequenced in paired-end mode on an Illumina MiSeq platform using the MiSeq Reagent Kit v3 (Illumina, Inc., San Diego, CA, USA), 2 × 300 bp chemistry [31,32].

Per-sample sequencing statistics, including raw read pairs, filtered reads, merged reads, chimera-free reads, mean merged read length, and final read depth used for downstream analyses, are provided in Supplementary Table S2.

After quality filtering, read merging, chimera removal, and removal of low-quality sequences, the final sequencing depth averaged approximately 45,000 reads per sample, with a range of approximately 30,000–60,000 reads.

### 2.4. Sequence Processing and Taxonomic Annotation

Raw paired-end FASTQ files were processed using QIIME 2 (v2023) and DADA2 (v2023.9). Primer sequences were removed using Cutadapt (v2023.9). Reads were quality filtered with maxN = 0, truncQ = 2, and expected-error filtering. Forward and reverse reads were truncated according to the quality-score profiles while maintaining sufficient overlap for merging of the V3–V4 amplicon. DADA2 was then used for dereplication, error-model learning, denoising, paired-end read merging, and chimera removal using the consensus method [33].

The analysis used amplicon sequence variants rather than 97% ASVs. DADA2 infers exact sequence variants by modeling and correcting Illumina amplicon errors and can resolve sequence differences as small as one nucleotide [33]. This approach was adopted to address the reviewer’s concern that 97% ASV clustering is outdated for modern microbiome analysis.

Representative ASV sequences were taxonomically assigned against the SILVA SSU rRNA reference database, release 138.1, using a Naive Bayes classifier trained for the 338F/806R V3–V4 region. Taxonomic assignment was performed with a confidence threshold of 0.70. Chloroplast, mitochondrial, archaeal, and unassigned non-bacterial sequences were removed before downstream bacterial community analysis [34].

For alpha- and beta-diversity analyses, samples were rarefied to an even depth of 30,000 sequences per sample, which retained all 18 biological samples. Rarefaction was used only for diversity estimation and ordination based on comparable sequencing depth. For abundance-based taxonomic summaries, correlation analyses, and predicted functional comparisons, non-rarefied ASV counts were normalized using relative abundance or appropriate transformed abundance tables [35,36].

### 2.5. Community Composition, Diversity, Environmental Association, and Functional Prediction

Alpha diversity was estimated using observed ASVs, ACE richness indices. Differences among sampling groups were tested using the Kruskal–Wallis test followed by Dunn’s post hoc test where appropriate. *p*-values from multiple pairwise tests were adjusted using the Benjamini–Hochberg false discovery rate procedure [36].

Beta diversity was calculated using Bray–Curtis dissimilarity and visualized by principal coordinate analysis. These analyses were performed in R (v4.6.0) using the vegan (v2.7-3) and phyloseq (v1.57.0) packages [37].

Community composition was summarized using stacked bar plots, heatmaps, Venn/UpSet diagrams, and Circos plots. Venn/UpSet analyses were based on ASVs or the lowest reliable taxonomic assignments, not on “species-level taxa.” Circos plots were used only for visualization of sample–taxon relationships.

Associations between soil physicochemical variables and bacterial taxa were evaluated using Spearman rank correlation analysis. Taxa included in correlation analysis were filtered to retain only taxa occurring in at least 20% of samples and with a mean relative abundance greater than 0.1%. Raw *p*-values were adjusted using the Benjamini–Hochberg false discovery rate procedure, and statistical significance was interpreted using FDR-adjusted *p*-values.

The relationship between the overall bacterial community structure and measured soil physicochemical variables was further evaluated using Mantel tests based on Bray–Curtis community dissimilarity and Euclidean environmental-distance matrices. Mantel results were interpreted conservatively, especially where correlations were weak or statistically non-significant [38].

Putative ecological functions were predicted from bacterial taxonomic profiles using FAPROTAX 1.2.12. FAPROTAX results are reported as predicted functional potential only, not as direct evidence of measured microbial activity. Differences in predicted functional categories among groups were tested using the Kruskal–Wallis test, with Benjamini–Hochberg FDR correction for multiple testing [39].

For PERMANOVA analysis, homogeneity of multivariate dispersion was checked using the betadisper function, because differences in dispersion may affect the interpretation of group differences.

Exploratory taxon-level association networks were constructed only as visual summaries of co-variation among abundant bacterial ASVs or lowest assigned taxa within each sampling group. Pairwise Spearman correlations were calculated using relative-abundance data after filtering low-abundance taxa. Retained positive and negative correlations were visualized as networks, and node/edge numbers and transitivity were reported descriptively [40,41,42]. The study included 18 samples in total, with three biological replicates in each of the six sampling groups, the statistical power to detect small differences among groups was limited; therefore, the results were interpreted cautiously as preliminary site-level patterns.

## 3. Results

### 3.1. Soil Physicochemical Properties of the Caspian Region

The measured soil physicochemical properties varied among the six sampling groups (Table 1). Values were recalculated and checked from the analytical dataset and are presented as mean ± SD based on three biological replicates per group; individual replicate values are provided in Supplementary Table S3. All soils were alkaline, with mean pH values ranging from 7.80 ± 0.10 in B2 to 9.00 ± 0.17 in B1. Soil organic matter, total nitrogen and total phosphorus were generally higher in B2 and M2 than in the other groups, whereas B1 showed the lowest values for these nutrient-related variables. The SD values for several B2 parameters, especially exchangeable Ca, exchangeable Mg, total nitrogen and total phosphorus, indicate within-group heterogeneity; therefore, B2 should not be interpreted as uniformly nutrient-rich across all replicates.

Exchangeable cations also differed among sampling groups. M2 showed the highest exchangeable Ca and Na, whereas B2 showed the highest exchangeable Mg. Available phosphate was highest in B2 and I1, while available K was highest in M1. These soil-property differences provide environmental context for interpreting the bacterial community data. However, because the Mantel relationship between the complete soil-property matrix and bacterial community structure was weak and non-significant, these physicochemical differences are interpreted as possible associations rather than direct explanatory drivers of the observed microbial patterns.

### 3.2. Bacterial Diversity and Community Composition Analysis

#### 3.2.1. Bacterial Diversity and Community Structure Across Sampling Sites

**Figure 1:**
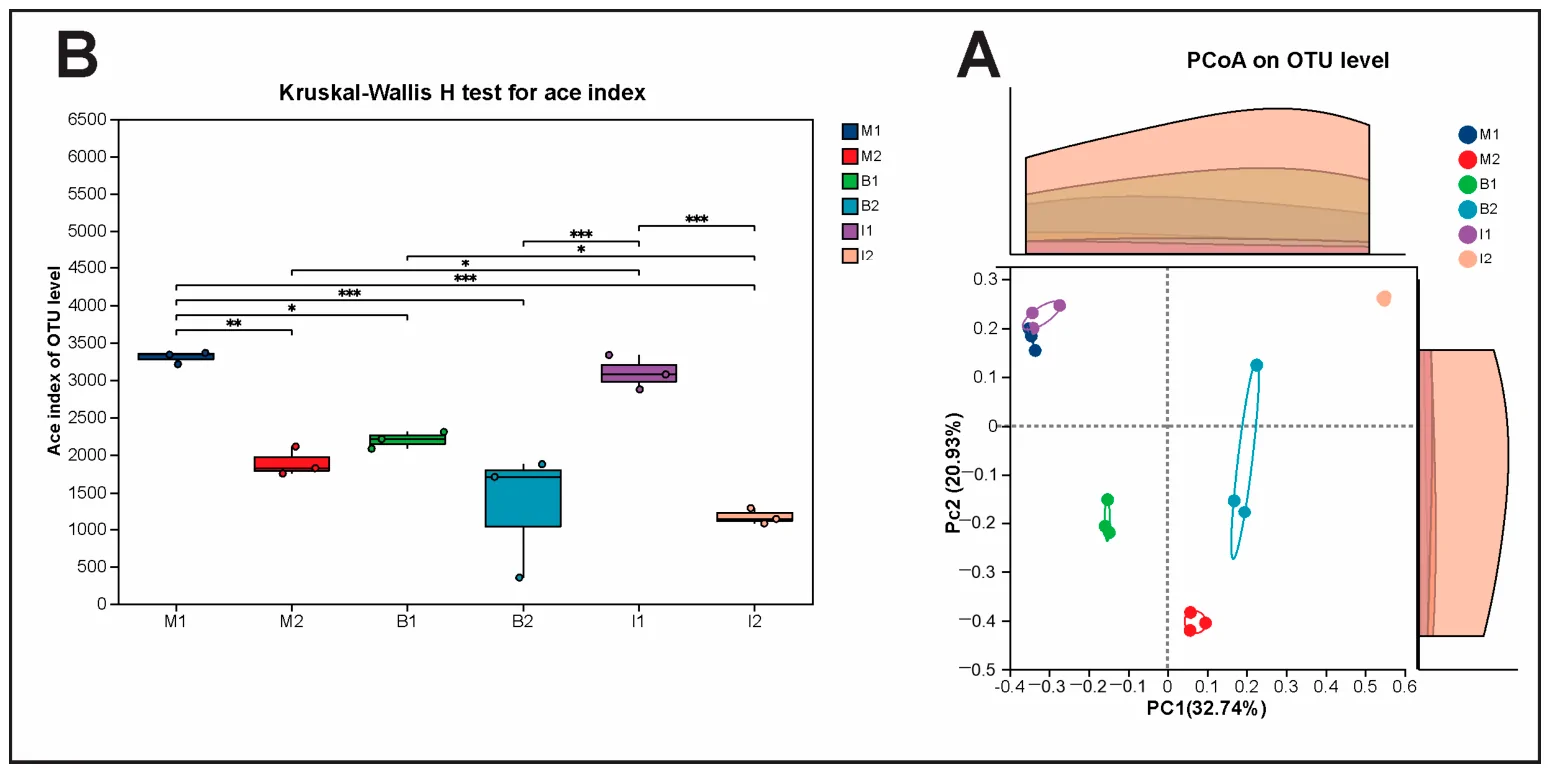
Alpha and beta diversity of soil bacterial communities. (**A**) ACE richness index showing differences in bacterial alpha diversity among M1, M2, B1, B2, I1 and I2. Significant differences were tested using the Kruskal–Wallis H test. (**B**) CoA based on ASV-level community composition showing beta-diversity separation between I1 and I2. Abbreviations: M1 and M2, Makat sampling sites; B1 and B2, Beyneu sampling sites; I1 and I2, Isatay sampling sites. Asterisks indicate statistical significance: * *p* < 0.05, ** *p* < 0.01, and *** *p* < 0.001.

Alpha diversity analysis based on the ACE richness index showed significant differences among the six soil groups (Kruskal–Wallis test, *p* = 0.00927). The effect size was estimated using epsilon-squared (ε^2^), indicating a [small/moderate/large] effect of sampling group on ACE richness (ε^2^ = [X]). The highest bacterial richness was observed in M1 and I1, with mean ACE values of approximately 3303 and 3094, respectively. B1 showed an intermediate richness level, followed by M2. In contrast, B2 and I2 had lower ACE values, with I2 showing the lowest richness among all groups. B2 also showed high variation among replicates, indicating stronger within-site heterogeneity. These results indicate that bacterial richness differed among the sampled soils; however, ecological explanations for these differences should be considered tentative because of the limited number of biological replicates per group (Figure 1).

Beta-diversity analysis based on Bray–Curtis dissimilarity and PCoA showed partial visual separation among sampling groups. However, because the first two PCoA axes represent only part of the total community variation, the ordination pattern was interpreted cautiously and supported by statistical testing rather than by visual clustering alone (Figure 1).

#### 3.2.2. Community Composition Analysis

The bacterial community composition of 18 soil samples from western Kazakhstan was summarized using genus-level heatmap clustering, genus-level community barplots, a genus-level Venn/UpSet analysis, and genus-level Circos analysis. The six groups represented three regions with three replicates per group: M1 and M2 from Makat, B1 and B2 from Beyneu, and I1 and I2 from Isatay. Unless explicitly supported by statistical testing, the following taxonomic comparisons are interpreted as descriptive patterns of relative abundance.

**Figure 2:**
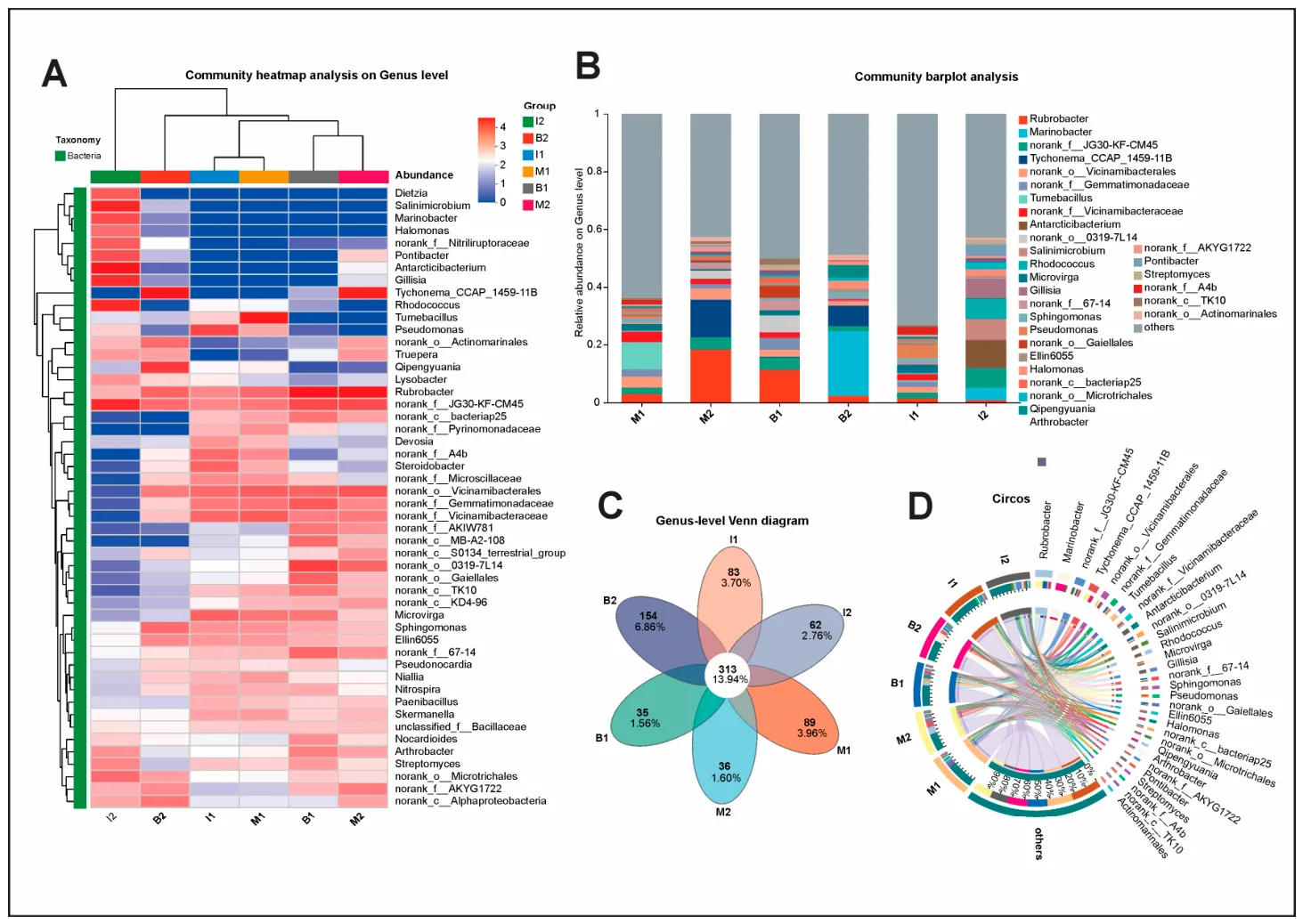
Community composition analysis of bacterial communities in soil samples from western Kazakhstan. (**A**) Genus-level heatmap showing abundance patterns and clustering of dominant bacterial genera among groups. (**B**) Genus-level community barplot showing the relative abundance of dominant bacterial genera. (**C**) Genus-level Venn diagram showing shared and unique bacterial taxa among the six sampling groups. (**D**) Genus-level Circos plot showing sample-genus relationships and the contribution of dominant genera to each sampling group. Abbreviations: M1, Makat region 1; M2, Makat region 2; B1, Beyneu region 1; B2, Beyneu region 2; I1, Isatay region 1; I2, Isatay region 2.

The genus-level heatmap showed differences in bacterial community composition among the six sampling groups (Figure 2A). The clustering pattern indicated that M1 was close to I1, while B1 clustered with M2, suggesting similar dominant genus-level profiles between these groups. In contrast, B2 formed a separate branch, and I2 was the most distinct group in this descriptive analysis. M1 and I1 were characterized by contributions from *Tumebacillus*, *Pseudomonas*, *Microvirga*, norank_f__*Vicinamibacteraceae*, and norank_o__*Vicinamibacterales*. B1 and M2 were mainly associated with *Rubrobacter*, *Tychonema*_CCAP_1459-11B, norank_o__0319-7L14, and norank_f__JG30-KF-CM45. I2 showed higher relative abundance of *Antarcticibacterium*, *Salinimicrobium*, *Rhodococcus*, *Gillisia*, *Marinobacter*, *Pontibacter*, *Dietzia*, and *Halomonas*.

The genus-level community barplot further showed variation in dominant bacterial taxa among the sampling groups (Figure 2B). In Makat, M1 was mainly characterized by *Tumebacillus*, unclassified *Vicinamibacterales*/*Vicinamibacteraceae*, *Rubrobacter*, and norank_f__*Gemmatimonadaceae*, whereas M2 showed high relative abundance of *Rubrobacter* and *Tychonema*_CCAP_1459-11B. In Beyneu, B1 showed high relative abundance of *Rubrobacter*, together with norank_o__0319-7L14, norank_f__*Gemmatimonadaceae*, norank_o__*Gaiellales*, and norank_f__JG30-KF-CM45. In contrast, B2 contained higher relative abundance of *Marinobacter*, with additional contributions from Tychonema_CCAP_1459-11B, *Qipengyuania*, *Halomonas*, and *Rubrobacter*. Because direct salinity measurements were not included, the occurrence of *Marinobacter* and *Halomonas* is discussed only as a taxonomic pattern consistent with possible stress- or salt-associated bacteria, not as evidence that salinity drove their occurrence. In Isatay, I1 showed a more even genus-level composition, while I2 was enriched in *Antarcticibacterium*, *Salinimicrobium*, *Rhodococcus*, *Gillisia*, norank_f__JG30-KF-CM45, *Marinobacter*, and *Pontibacter*.

The genus-level Venn/UpSet analysis revealed both shared and unique bacterial taxa among the six sampling groups (Figure 2C). A total of 313 genus-level taxa were shared by all groups, accounting for 13.94% of the detected taxa, indicating the presence of a common core community across the investigated soils. Each group also contained unique taxa, showing local differentiation. B2 contained the highest number of unique taxa, with 154 taxa, followed by M1 with 89, I1 with 83, I2 with 62, M2 with 36, and B1 with 35 unique taxa. Total richness in the Venn/UpSet analysis was highest in M1 and I1, with 1471 and 1458 taxa, respectively, followed by B2, B1, M2, and I2 with 1174, 982, 975, and 845 taxa, respectively. These results indicate higher taxonomic richness in M1 and I1 and a larger number of unique taxa in B2.

The genus-level Circos analysis illustrated the relationship between sample groups and dominant genera (Figure 2D). The “others” fraction represented the largest proportion in all groups, indicating that many low-abundance genera contributed to the bacterial communities. B2 showed a strong association with *Marinobacter*, together with Tychonema_CCAP_1459-11B, *Qipengyuania*, *Halomonas*, and *Rubrobacter*. M2 was mainly linked with Rubrobacter and Tychonema_CCAP_1459-11B. B1 was associated with *Rubrobacter*, norank_o__0319-7L14, norank_f__*Gemmatimonadaceae*, norank_o__*Gaiellales*, and norank_f__JG30-KF-CM45. M1 was mainly connected with *Tumebacillus*, norank_o__*Vicinamibacterales*, norank_f__*Vicinamibacteraceae*, and *Rubrobacter*. I2 showed links with *Antarcticibacterium*, *Salinimicrobium*, *Rhodococcus*, *Gillisia*, norank_f__JG30-KF-CM45, *Marinobacter*, and *Pontibacter*, while I1 had weaker links to individual dominant genera, consistent with its more even community composition.

Although the discussion focuses mainly on dominant genera, rare taxa may also have ecological significance in arid soils, because low-abundance microorganisms can contribute to functional resilience, stress response, and community recovery under changing environmental conditions.

Together, these analyses show spatial heterogeneity in the soil bacterial communities. The heatmap and barplot showed that dominant genera differed among sampling groups, while the Venn/UpSet analysis confirmed the presence of both shared and site-specific taxa. The Circos plot further supported these patterns by identifying sample-genus relationships. These results are interpreted as descriptive evidence of spatial variation in taxonomic composition rather than proof that the measured soil variables caused the observed differences.

### 3.3. Associations Between Soil Properties, Bacterial Taxa and Predicted Functions

**Figure 3:**
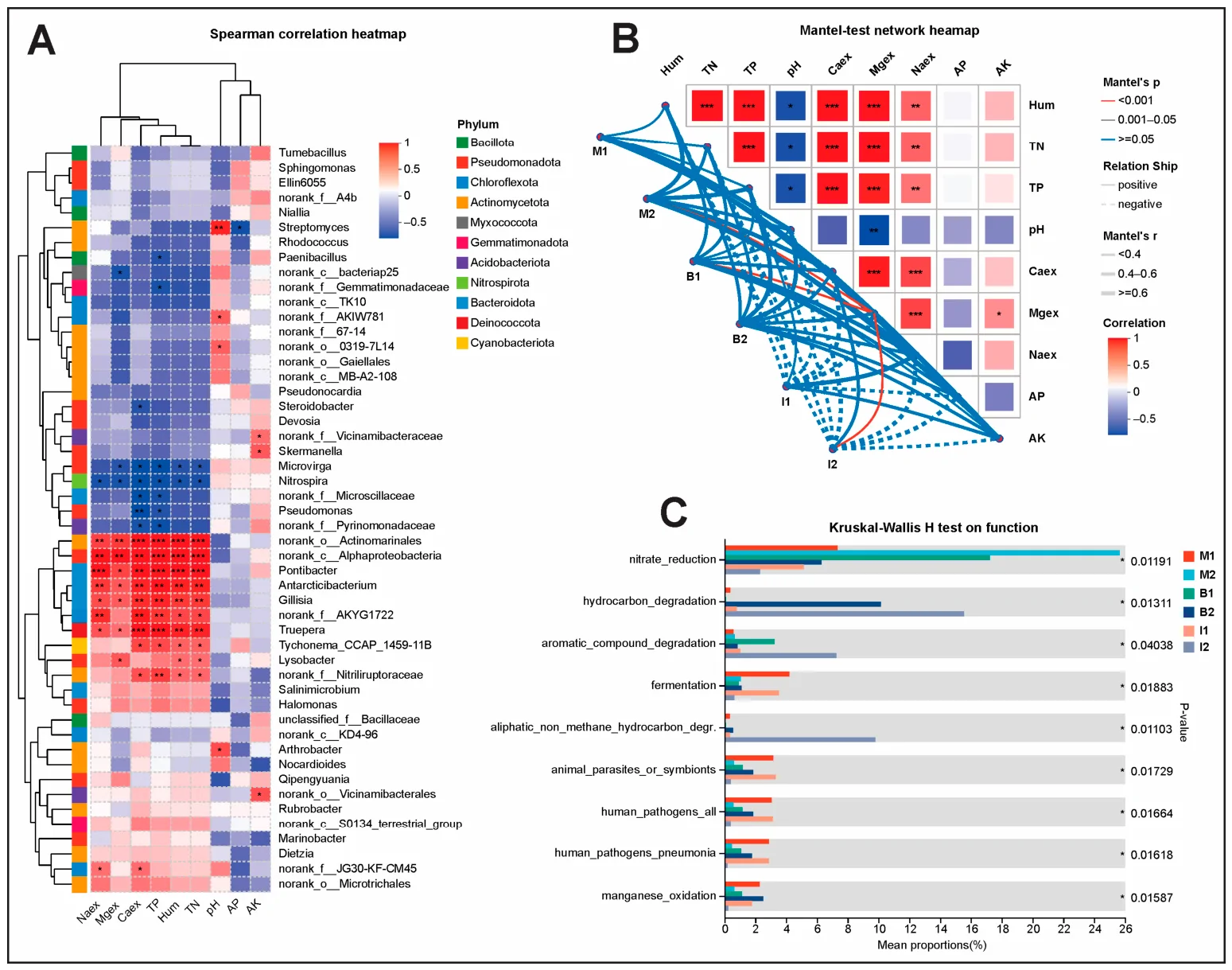
Relationships between soil physicochemical properties, bacterial community structure, and predicted functions. (**A**) Correlation between soil physicochemical properties and dominant bacterial taxa. (**B**) Relationships between soil physicochemical properties and bacterial community structure shown by Mantel-test network heatmap. (**C**) Differential prediction of bacterial functional profiles among sampling groups. Abbreviations: M1, Makat region 1; M2, Makat region 2; B1, Beyneu region 1; B2, Beyneu region 2; I1, Isatay region 1; I2, Isatay region 2. Red indicates positive correlations, blue indicates negative correlations, and asterisks indicate statistically significant correlations in the correlation heatmap. Hum, soil organic matter; TN, total nitrogen; TP, total phosphorus; Caex, exchangeable calcium; Mgex, exchangeable magnesium; Naex, exchangeable sodium; AP, available phosphorus; AK, available potassium. Asterisks indicate statistical significance: * *p* < 0.05, ** *p* < 0.01, and *** *p* < 0.001.

The dominant bacterial taxa included in the correlation heatmap showed positive and negative associations with soil physicochemical properties (Figure 3A). Positive correlations with soil organic matter, TN, TP, Caex, Mgex, and Naex were observed for taxa such as norank_o__*Actinomarinales*, norank_c__*Alphaproteobacteria*, *Pontibacter*, *Truepera*, *Antarcticibacterium*, *Gillisia*, and norank_f__AKYG1722. Among them, norank_o__*Actinomarinales*, norank_c__*Alphaproteobacteria*, *Pontibacter*, and *Truepera* showed consistent positive associations across multiple soil variables. In contrast, *Nitrospira*, *Microvirga*, *Pseudomonas*, norank_f__*Microscillaceae*, norank_f__*Pyrinomonadaceae*, norank_f__*Gemmatimonadaceae*, *Paenibacillus*, and *Steroidobacter* were negatively associated with several nutrient-related properties. The pattern for pH differed from that of nutrient variables, with positive correlations mainly observed for Streptomyces, Arthrobacter, norank_o__0319-7L14, and norank_f__AKIW781. Available K showed a weaker and more selective pattern, being positively associated mainly with norank_o__*Vicinamibacterales*, norank_f__*Vicinamibacteraceae*, and *Skermanella*, whereas available phosphorus showed only limited associations. These results indicate associations between soil chemical variables and bacterial taxa, they do not demonstrate that these variables directly favored, selected, or drove the occurrence of these taxa.

The Mantel-test network showed that the measured soil variables were themselves strongly interrelated (Figure 3B). Soil organic matter and TN were perfectly positively correlated (r = 1.000), TP was also strongly correlated with soil organic matter and TN, and Caex and Mgex were positively associated with these nutrient variables, whereas pH tended to be negatively related to soil organic matter, TN, TP, and Mgex. However, despite these environmental correlations, the overall Mantel relationship between soil properties and bacterial community structure was weak and not significant (r = 0.0376, *p* = 0.30). Therefore, the measured soil variables should not be described as significant drivers of overall bacterial community structure in this dataset.

FAPROTAX analysis predicted differences in potential functional categories among sampling groups (Figure 3C). These results represent taxonomically inferred functional potential rather than directly measured microbial activities. The Kruskal–Wallis test identified 15 predicted categories with between-group variation at the *p*-value level. The strongest differences were observed for aromatic hydrocarbon degradation (*p* = 0.00715), aliphatic non-methane hydrocarbon degradation (*p* = 0.01103), nitrate reduction (*p* = 0.01191), nitrogen respiration (*p* = 0.0128), and hydrocarbon degradation (*p* = 0.01311). Predicted hydrocarbon-related categories were relatively higher in Isatay- and B2-associated communities, whereas predicted nitrate reduction was higher in M2 and B1. Fermentation, ureolysis, manganese oxidation, dark oxidation of sulfur compounds, and several host-associated/pathogen-related FAPROTAX categories also varied among groups. The pathogen-related categories are reported only as unvalidated taxonomic predictions.

### 3.4. Exploratory ASV/Taxon-Level Association Network Analysis

**Figure 4:**
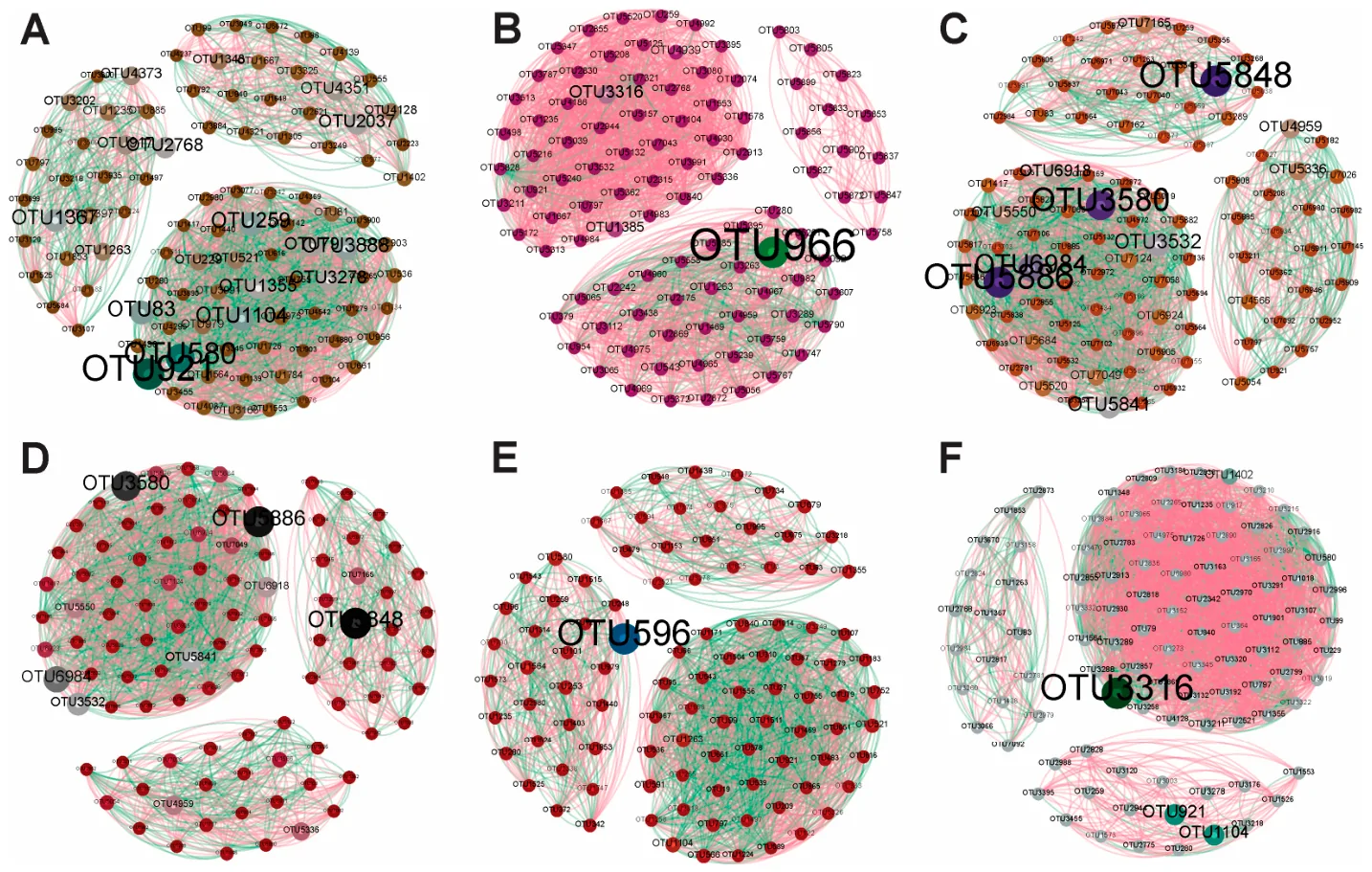
Exploratory ASV/taxon-level association network visualization of dominant bacterial taxa. (**A**) B1, (**B**) B2, (**C**) I1, (**D**) I2, (**E**) M1, and (**F**) M2. Abbreviations: M1, Makat region 1; M2, Makat region 2; B1, Beyneu region 1; B2, Beyneu region 2; I1, Isatay region 1; I2, Isatay region 2. Red edges indicate positive Spearman correlations, whereas green edges indicate negative Spearman correlations. Node labels represent ASV/taxon IDs. Because of the low number of biological replicates per group and the compositional nature of the data, the retained links should be interpreted as exploratory association patterns rather than direct biological interactions.

Species-level co-occurrence correlation networks were used as exploratory visual summaries of co-variation among dominant ASV/ASVs annotated taxa in each sampling group (Figure 4). Because each group contained only three biological replicates and the data are compositional 16S rRNA amplicon data, the networks should not be interpreted as stable site-specific interaction networks. Instead, they indicate which dominant taxa were connected by retained positive or negative Spearman correlations under the selected filtering criteria. All networks showed high clustering, with transitivity = 1.0, and were organized into disconnected modules rather than one continuous network. The number of retained edges varied among groups: M2 showed 100 nodes and 2369 edges, B2 showed 98 nodes and 1872 edges, B1, I1 and I2 each contained 99 nodes and 1827 edges, and M1 contained 98 nodes and 1758 edges.

In the B1 exploratory network, many retained correlations involved *Actinomycetota*-associated ASVs, including *Rubrobacter* sp., *Rubrobacter*-affiliated uncultured bacteria, and unclassified *Rubrobacter* (Figure 4A). Other abundant or connected B1 taxa included *Arthrobacter*, *Gaiellales* bacterium, 0319-7L14 bacterium, *Gemmatimonadaceae* bacterium, JG30-KF-CM45 bacterium, and *Nocardioides albus*. In B2, the retained correlations involved *Marinobacter*, *Tychonema*_CCAP_1459-11B, *Qipengyuania*, *Nodosilinea*_PCC-7104, *Blastocatellaceae* bacterium, *Pseudonocardia*, Ellin6055, *Halomonas zincidurans*, and Halomonas ventosae (Figure 4B). The occurrence of *Marinobacter* and *Halomonas* is consistent with the taxonomic composition results, but it should not be interpreted as direct evidence of salinity-driven interactions.

The Isatay exploratory networks were characterized by different dominant species-level ASVs. I1 included taxa assigned to *Rhodococcus*, *Gillisia*, *Antarcticibacterium*, *Salinimicrobium*, JG30-KF-CM45 bacterium, *Marinobacter*, *Dietzia psychralcaliphila*, and *Pontibacter* (Figure 4C). A similar set of taxa was observed in I2, where *Gillisia*, *Antarcticibacterium*, *Salinimicrobium*, JG30-KF-CM45 bacterium, *Marinobacter*, *Dietzia psychralcaliphila*, and *Pontibacter* were connected by retained correlations (Figure 4D). In I2, *Rhodococcus* was abundant but showed lower connectivity than several other taxa.

The Makat exploratory networks also differed between the two sampling sites. In M1, *Tumebacillus* was the most abundant species-level ASV, while retained correlations also involved *Rubrobacter*, Ellin6055, *Microvirga*, *Niallia*, *Neobacillus*, *Gemmatimonadaceae* bacterium, *Skermanella*, *Priestia*, *Candidatus Udaeobacter*, and JG30-KF-CM45 bacterium (Figure 4E). In M2, abundant or connected taxa included Tychonema_CCAP_1459-11B, several *Rubrobacter*-related ASVs, *Actinomarinales* bacterium, JG30-KF-CM45 bacterium, *Gemmatimonadaceae* bacterium, *Euzebyaceae* bacterium, 0319-7L14 bacterium, and *Truepera* (Figure 4F). The larger number of retained edges in M2 is reported as a descriptive topological feature only.

Exploratory ASV/taxon-level association networks were used as visual summaries of co-variation among dominant ASVs or lowest-rank annotated taxa in each sampling group (Figure 4). Because each group contained only three biological replicates and the data are compositional 16S rRNA amplicon data, the networks should not be interpreted as stable site-specific interaction networks. Instead, they indicate which dominant ASVs/taxa were connected by retained positive or negative Spearman correlations under the selected filtering criteria. All networks showed high clustering, with transitivity = 1.0, and were organized into disconnected correlation clusters rather than one continuous network. The number of retained edges varied among groups: M2 showed 100 nodes and 2369 edges, B2 showed 98 nodes and 1872 edges, B1, I1 and I2 each contained 99 nodes and 1827 edges, and M1 contained 98 nodes and 1758 edges. These differences are presented descriptively and should not be used to infer stronger ecological interaction or network stability.

## 4. Discussion

This study provides an integrated 16S rRNA-based assessment of soil bacterial communities in dryland soils from the western Kazakhstan Caspian region, including sites in the Makat and Isatay districts of Atyrau Region and the Beyneu district of Mangystau Region. The region is ecologically important because it combines aridity, alkaline soils, sparse vegetation, salt-affected landscapes and intensive land use associated with oil and gas development. However, the present study did not directly measure petroleum hydrocarbons, PAHs, total soluble salts, electrical conductivity or detailed vegetation and moisture parameters. Therefore, the discussion below interprets the microbial patterns as associations observed within the sampled soils and as hypotheses for future testing, rather than as direct evidence of particular contamination sources or measured functional activity [43,44,45].

The broader value of these data is that dryland and semi-desert microbiomes of Central Asia remain less intensively studied than those of several other arid regions. Previous studies from Kazakhstan and neighboring arid environments have shown that dry soils can contain distinctive bacterial assemblages, including actinobacteria and other stress-tolerant groups, and that oil-affected or saline soils may harbor taxa with biotechnological relevance [46]. Compared with these earlier reports, our work contributes a site-level comparison from the western Caspian dryland sector by combining soil physicochemical measurements, alpha and beta diversity, taxonomic composition, taxon-environment correlations, FAPROTAX-based functional prediction and exploratory co-occurrence networks. The design is still limited by the small number of replicate samples per group; consequently, the main contribution of the study is to identify descriptive microbial patterns and candidate taxa that warrant more detailed ecological, chemical and functional validation.

### 4.1. Shared and Site-Specific Bacterial Patterns Across the Sampled Soils

The most robust community-level pattern was the coexistence of shared taxa across the sampled sites with marked site-level differentiation. A total of 313 taxa were detected in all six sampling groups, indicating that some bacterial taxa were common across the investigated soils. Because the study included only six groups and 18 samples, this result should not be described as evidence of a stable regional core microbiome. Rather, it shows that shared taxa were detected across the sampled sites, while each group also contained a distinct local component. This more conservative interpretation is consistent with broader studies showing that dryland and alkaline soils often contain recurrent bacterial lineages but can also display strong habitat-specific turnover at local scales [47,48].

The alpha and beta diversity results strengthen this interpretation. ACE richness differed significantly among the six groups, with M1 and I1 showing the highest richness and I2 showing the lowest richness, whereas the PCoA analysis showed clear separation between I1 and I2. Thus, even geographically related soils can differ strongly in community structure. The genus-level heatmap, barplot, Venn analysis and Circos plot further showed that B1 and M2 were mainly associated with Rubrobacter-related and other actinobacterial taxa, B2 was enriched in *Marinobacter*-, *Halomonas*- and *Qipengyuania*-containing assemblages, and I2 had a distinct composition that included *Antarcticibacterium*, *Salinimicrobium*, *Rhodococcus*, *Gillisia*, *Pontibacter*, *Dietzia* and *Marinobacter* (Figure 1 and Figure 2).

Several aspects of these patterns agree with comparative dryland microbiome studies. Actinomycetota and related drought-tolerant taxa are frequently reported from arid and semi-arid soils, while habitat differences in pH, moisture, salinity, vegetation and nutrient availability can produce strong beta-diversity shifts [49,50]. Studies of Kazakhstan desert soils have also emphasized the presence of distinctive actinobacterial communities and the potential biotechnological value of dryland microorganisms [25,26]. At the same time, the western Caspian region has specific ecological features, including strong continental aridity, salt-affected landscapes and oilfield-associated land use, which may contribute to local microbial differentiation.

### 4.2. Soil Physicochemical Properties and the Non-Significant Mantel Result

The measured soil properties provided useful environmental context for interpreting the bacterial data. All soils were alkaline, but they differed in organic matter, total nitrogen, total phosphorus, exchangeable cations and available phosphorus and potassium. Spearman correlations suggested that several genera were associated with nutrient- and cation-related variables, whereas other taxa were more closely associated with higher pH or lower nutrient conditions. These correlations are useful for describing taxon-environment associations, but they should not be interpreted causally. Relative-abundance correlations do not demonstrate that soil variables drove the occurrence of individual taxa, and the small sample size further limits inference (Figure 3A) [51,52,53].

The Mantel test is especially important for interpreting the study. Although individual soil variables were strongly correlated with one another, the overall relationship between the complete soil-property matrix and bacterial community structure was weak and non-significant (r = 0.0376, *p* = 0.30). Therefore, the measured physicochemical variables cannot be presented as statistically supported drivers of the total bacterial community structure in this dataset. The more appropriate conclusion is that the measured soil properties may help contextualize some observed microbial patterns, but they do not explain the overall community dissimilarity at the level tested by the Mantel analysis (Figure 3B).

This non-significant Mantel result should be treated as a design limitation rather than as evidence for a confirmed multicausal assembly process. Many potentially important variables were not measured, including electrical conductivity, total soluble salts, chloride, sulfate, carbonate content, soil texture, water content, vegetation cover, root influence, recent wetting history, petroleum hydrocarbons, PAHs and trace metals. Any of these factors could contribute to microbial community variation in Caspian dryland soils. Thus, our interpretation is that bacterial communities differed among the sampled soils, but the present design cannot fully resolve the environmental causes of those differences. Future studies should combine larger spatial replication with direct salinity, hydrocarbon, metal, moisture and vegetation measurements to test the mechanisms suggested by the current descriptive patterns [48].

The weak and non-significant Mantel relationship may reflect the limited sample size and the restricted set of measured soil variables. Important unmeasured factors, including soil moisture, salinity indicators, soil texture, vegetation cover, petroleum hydrocarbons, PAHs, and trace metals, may also contribute to bacterial community variation in these dryland soils.

### 4.3. Conservative Interpretation of Stress-Associated and Hydrocarbon-Related Taxa

Several dominant taxa were consistent with bacterial groups commonly reported from dry, alkaline or otherwise stressful soils. *Rubrobacter*-related ASVs were prominent in B1 and M2, while *Truepera*, *Pontibacter*, *Arthrobacter*, *Streptomyces*, *Tumebacillus* and related groups were also detected among important taxa. These genera include members known from arid, radiation-exposed, alkaline, saline or nutrient-limited environments, and their presence is compatible with the dryland setting of western Kazakhstan [54]. However, this interpretation should remain ecological and comparative: detection of such taxa indicates that the communities contained lineages with known stress-tolerant representatives, but it does not directly prove physiological adaptation in the specific strains present in our samples.

The interpretation of *Marinobacter*, *Halomonas*, *Rhodococcus* and *Dietzia* also requires caution. These genera contain species that have been reported from saline, oil-affected or hydrocarbon-contaminated environments, and FAPROTAX predicted functions related to aromatic and aliphatic hydrocarbon degradation in some groups. Therefore, it is reasonable to describe B2 and Isatay communities as containing taxa with representatives known from hydrocarbon-affected environments and as showing predicted hydrocarbon-related functional potential. However, because total petroleum hydrocarbons, PAHs and oil content were not measured in the sampled soils, these communities should not be described as hydrocarbon-rich, oil-contaminated or actively hydrocarbon-transforming on the basis of the present data alone [55].

Similarly, the occurrence of *Marinobacter*, *Halomonas*, *Salinimicrobium*, *Gillisia* and *Antarcticibacterium* may be consistent with saline or mineralized environments, but direct salinity indicators were not measured. Exchangeable Na alone is insufficient to define soil salinity status. Electrical conductivity, total soluble salts, chloride, sulfate and related parameters would be necessary to confirm salinity gradients [56].

These taxon–soil correlations indicate associations only and should not be interpreted as causal evidence that the measured soil properties directly controlled bacterial community composition.

### 4.4. Functional Prediction as Hypothesis Rather than Measured Activity

The FAPROTAX results should be interpreted as predicted functional profiles derived from taxonomic affiliation, not as measured soil functions. The observed differences in predicted nitrate reduction, nitrogen respiration, fermentation, ureolysis, sulfur-related processes and hydrocarbon degradation are useful for hypothesis generation, but they do not demonstrate actual process rates, gene abundance, transcript expression or biochemical activity. This distinction is important because 16S-based functional inference has limited resolution and depends on the availability and ecological accuracy of reference annotations [57].

For the same reason, interpretations related to dryland pulse-response strategies, nitrogen transformations, cyanobacterial carbon fixation and biocrust functions are presented here only as functional hypotheses. Dryland literature shows that microbial processes can respond rapidly to wetting events and that cyanobacteria in biological soil crusts can contribute to carbon fixation, nitrogen cycling and surface stabilization [58]. In our dataset, cyanobacterial taxa and predicted nitrogen-related functions suggest that such processes may be relevant in western Kazakhstan dryland soils, but the present study did not measure wetting-response rates, functional genes, transcripts, carbon fixation, nitrogen fixation or biocrust activity. These hypotheses should be tested using controlled wetting incubations, quantitative functional-gene assays, metagenomics, metatranscriptomics, process-rate measurements and field observations of biocrust development [59].

Because petroleum hydrocarbons, PAHs, and other soil pollutants were not directly measured, FAPROTAX-predicted hydrocarbon-related functions should be interpreted only as putative functional potential, not as evidence of active hydrocarbon degradation or bioremediation capacity.

### 4.5. Exploratory Network Analysis and Practical Implications

The ASV/taxon-level association networks should be considered exploratory visualizations of association patterns among abundant ASVs or lowest assigned taxa. With only three biological replicates per group, the networks cannot reliably establish stable site-specific interaction structures, direct microbial interactions, exclusion relationships, or statistically robust co-occurrence modules. Apparent co-variation can arise from shared habitat preference, compositional effects, or other statistical artifacts, and it does not prove trophic, syntrophic, or antagonistic interactions. Therefore, the networks are useful mainly for identifying dominant taxa that co-varied within each group and for generating hypotheses about local community organization. Stronger network inference would require larger sample numbers, compositional-data-aware methods, independent validation, and ideally temporal or experimental datasets (Figure 4).

The practical significance of the detected bacterial communities should also be framed cautiously. The present work did not isolate strains, test hydrocarbon degradation, measure plant-growth promotion, conduct greenhouse or field bioinoculation experiments, or validate microbiome engineering approaches. Therefore, taxa such as *Rubrobacter*, *Truepera*, *Pontibacter*, *Marinobacter*, *Halomonas*, *Rhodococcus*, *Dietzia* and cyanobacterial lineages should be regarded as candidate taxa for future isolation, cultivation and functional testing rather than as confirmed bioinoculants, bioremediation agents or restoration tools.

Practical significance of bacterial communities may also be considered in a larger context of sustainable soil and crop management practices. Recently, it has been revealed that fertilizer application regimes substantially affect both agrochemical characteristics of soil and its productivity [60], nitrogen-fixing bacteria and humic substances synthesized from agricultural wastes have also been suggested as promising tools for improvement of soil fertility and biological sustainability in soybean cultivation [60,61]. Moreover, seed inoculation with bacterial biological preparations and foliar application of growth-stimulating substances have been demonstrated to enhance growth and yields of organically grown soybeans [62]. These examples show that microbial and organic amendments can be relevant for sustainable soil management, but analogous applications in Caspian dryland soils will require local strain isolation, functional screening, safety assessment and field validation.

## 5. Conclusions

This study characterized soil bacterial communities from six dryland sampling groups in the Caspian region of western Kazakhstan using soil physicochemical measurements and 16S rRNA gene sequencing. The sampled soils were alkaline and chemically heterogeneous, and bacterial communities differed among sites in richness, composition and dominant taxa. Shared taxa were detected across all sampled sites, but each group also showed site-specific taxonomic patterns. M1 and I1 showed higher bacterial richness, B2 contained the highest number of unique taxa, and I2 was compositionally distinct from I1. Associations between soil variables and bacterial taxa were observed, but these should be interpreted cautiously. The Mantel test showed a weak and non-significant relationship between the measured soil-property matrix and overall bacterial community structure. Therefore, the measured soil variables are best considered as contextual factors and possible contributors to microbial variation, not as statistically confirmed drivers of community assembly. Important unmeasured variables, including electrical conductivity, soluble salts, chloride, sulfate, soil texture, moisture, vegetation, petroleum hydrocarbons, PAHs and trace metals, should be included in future studies. The detected communities included taxa with representatives known from dry, alkaline, saline, mineralized or hydrocarbon-affected environments, including *Rubrobacter*, *Tumebacillus*, *Marinobacter*, *Halomonas*, *Rhodococcus*, *Pontibacter*, *Gillisia*, *Salinimicrobium*, *Antarcticibacterium* and *Dietzia*. However, the present study did not directly measure salinity, petroleum hydrocarbons or hydrocarbon degradation. Accordingly, these taxa should be described as lineages with potential ecological relevance and as candidates for future isolation and functional testing, rather than as proof of salinity-driven or hydrocarbon-driven community assembly. Importantly, the functional features identified by FAPROTAX represent predicted potential based on taxonomic composition and do not provide experimental evidence of actual microbial metabolic activity. Exploratory association networks further highlighted dominant taxa that co-varied within sampling groups, but these patterns require validation with larger sample numbers and compositionality-aware network methods. Future work should combine larger sampling designs with raw-sequence deposition, complete metadata, ASV tables, representative sequences, chemical analyses, metagenomics or metatranscriptomics, cultivation, process-rate measurements and field validation.

## Figures and Tables

**Table 1:** Soil physicochemical properties of the sampling groups.

Group	Soil Organic Matter (%)	TN (%)	TP (%)	pH (1:2.5 H_2_O)	Caex (cmol(+) kg^−1^)	Mgex (cmol(+) kg^−1^)	Naex (cmol(+) kg^−1^)	Available Phosphorus (mg 100 g^−1^)	Available K (mg 100 g^−1^)
M1	0.38 ± 0.12	0.025 ± 0.006	0.037 ± 0.012	8.33 ± 0.06	8.00 ± 0.80	1.60 ± 0.00	0.404 ± 0.429	0.36 ± 0.15	52.48 ± 2.44
M2	1.19 ± 0.31	0.069 ± 0.018	0.110 ± 0.020	8.65 ± 0.05	11.60 ± 0.40	1.60 ± 0.00	0.875 ± 0.425	0.87 ± 0.34	37.56 ± 0.84
B1	0.14 ± 0.09	0.009 ± 0.006	0.023 ± 0.006	9.00 ± 0.17	2.80 ± 0.80	0.40 ± 0.00	0.063 ± 0.006	0.84 ± 0.66	9.84 ± 3.74
B2	2.39 ± 0.04	0.129 ± 0.158	0.113 ± 0.076	7.80 ± 0.10	9.33 ± 7.21	1.87 ± 1.22	0.223 ± 0.190	2.00 ± 1.04	12.32 ± 1.34
I1	0.22 ± 0.08	0.015 ± 0.005	0.023 ± 0.006	8.80 ± 0.26	1.47 ± 0.23	0.67 ± 0.23	0.095 ± 0.059	1.82 ± 0.94	27.36 ± 6.89
I2	0.69 ± 0.21	0.041 ± 0.011	0.077 ± 0.025	8.70 ± 0.17	9.60 ± 0.80	1.60 ± 0.00	0.465 ± 0.401	0.40 ± 0.13	30.96 ± 5.36

## Data Availability

The data supporting this study are available from the corresponding author upon reasonable request. PRJNA1473722. http://www.ncbi.nlm.nih.gov/bioproject/1473722 (accessed on 2 June 2026).

## Supplementary Materials

The following supporting information can be downloaded at: https://www.mdpi.com/article/10.3390/biology15120969/s1. Table S1: Site metadata for soil sampling groups in the Caspian drylands of western Kazakhstan; Table S2: Individual replicate values; Table S3: Per-sample sequencing statistics for bacterial 16S rRNA gene V3-V4 amplicon sequencing.

## Abbreviations

The following abbreviations are used in this manuscript:

16S rRNA	16S ribosomal ribonucleic acid
ASV	Amplicon sequence variant
DNA	Deoxyribonucleic acid
PCR	Polymerase chain reaction
OTU	Operational taxonomic unit
FAPROTAX	Functional Annotation of Prokaryotic Taxa
TN	Total nitrogen
TP	Total phosphorus
AP	Available phosphorus
AK	Available potassium
pH	Measure of soil acidity or alkalinity
