# Supplementary material for: Soil Bacterial Community Structure and Functional Potential in the Caspian Drylands of Western Kazakhstan
Source: Biology (Basel). 2026 Jun 20;15(12):969. doi: 10.3390/biology15120969 (PMC13296076; doi:10.3390/biology15120969)
Supplement: Supplementary file 1 [file biology-15-00969-s001.zip › Supplementary Table S2.pdf]

Supplementary Table S2. Individual replicate values used to calculate Table 1.

Group	Sample ID	SOM (%)	TN (%)	TP (%)	pH (1:2.5 H2O)	Caex (meq/100 g)	Mgex (meq/100 g)	Naex (meq/100 g)	AP (mg/100 g)	AK (mg/100 g)
M1	A1_MO_1	0.518	0.032	0.05	8.3	8.8	1.6	0.1532	0.53	41.52
M1	A1_MO_2	0.3108	0.021	0.03	8.4	8	1.6	0.9	0.27	66
M1	A1_MO_3	0.3108	0.021	0.03	8.3	7.2	1.6	0.1592	0.27	49.92
M2	A2_MA_1	1.5022	0.086	0.13	8.7	12	1.6	0.45	1.2	38.4
M2	A2_MA_2	1.1914	0.0685	0.11	8.65	11.6	1.6	0.875	0.86	37.56
M2	A2_MA_3	0.8806	0.051	0.09	8.6	11.2	1.6	1.3	0.53	36.72
B1	B1_MB_1	0.2415	0.0154	0.03	8.8	3.6	0.4	0.0582	1.6	14.16
B1	B1_MB_2	0.1035	0.007	0.02	9.1	2.8	0.4	0.0598	0.53	7.68
B1	B1_MB_3	0.069	0.0049	0.02	9.1	2	0.4	0.0696	0.4	7.68
B2	B2_BO_1	2.35	0.31	0.18	7.7	16.8	3.2	0.428	3.2	10.98
B2	B2_BO_2	2.39	0.06	0.13	7.8	8.8	1.6	0.19	1.47	12.32
B2	B2_BO_3	2.43	0.0168	0.03	7.9	2.4	0.8	0.0516	1.33	13.66
I1	C1_II_1	0.3108	0.021	0.03	8.5	1.6	0.8	0.0516	2.8	20.47
I1	C1_II_2	0.2072	0.014	0.02	8.9	1.6	0.8	0.162	1.73	27.36
I1	C1_II_3	0.1554	0.0105	0.02	9	1.2	0.4	0.0714	0.93	34.25
I2	C1_IK_1	0.7252	0.043	0.08	8.8	9.6	1.6	0.2008	0.53	25.60
I2	C1_IK_2	0.8806	0.051	0.1	8.8	10.4	1.6	0.2684	0.4	30.96
I2	C1_IK_3	0.4662	0.029	0.05	8.5	8.8	1.6	0.926	0.27	36.32
