# Supplementary material for: Soil Bacterial Community Structure and Functional Potential in the Caspian Drylands of Western Kazakhstan
Source: Biology (Basel). 2026 Jun 20;15(12):969. doi: 10.3390/biology15120969 (PMC13296076; doi:10.3390/biology15120969)
Supplement: Supplementary file 1 [file biology-15-00969-s001.zip › Supplementary Table S3.pdf]

Supplementary Table S3. Per-sample sequencing statistics for bacterial 16S rRNA gene V3-V4 amplicon sequencing

This table reports sequencing output after demultiplexing and quality control for the 18 composite soil samples used in the study. Raw reads are shown separately from merged, non-chimeric optimized sequences to avoid direct comparison of counts generated at different processing stages. The final rarefaction depth used for alpha- and beta-diversity analyses was 50,000 sequences per sample; all samples were retained.

Sample ID	Group	Biological replicate	Raw reads (provider report)	Filtered / clean reads	Merged reads / tags	Non-chimeric optimized sequences	Mean optimized length (bp)	Final depth used for diversity analyses	Retention from raw to optimized (%)
A1_MO_1	M1	1	75,855	72,446	70,689	67,589	417.4	50,000	89.1
A1_MO_2	M1	2	67,203	63,851	62,112	60,220	417.9	50,000	89.6
A1_MO_3	M1	3	65,788	62,490	60,379	59,732	417.8	50,000	90.8
A2_MA_1	M2	1	77,528	74,068	71,724	68,413	417.6	50,000	88.2
A2_MA_2	M2	2	69,885	66,816	65,537	61,801	416.2	50,000	88.4
A2_MA_3	M2	3	69,391	65,847	64,431	60,861	418.5	50,000	87.7
B1_MB_1	B1	1	69,036	65,688	64,499	60,774	416.9	50,000	88.0
B1_MB_2	B1	2	67,665	65,442	63,493	59,298	418.6	50,000	87.6
B1_MB_3	B1	3	77,445	75,125	73,648	68,283	417.3	50,000	88.2
B2_BO_1	B2	1	67,058	63,701	62,055	60,020	419.0	50,000	89.5
B2_BO_2	B2	2	76,466	73,882	72,733	69,777	418.1	50,000	91.3
B2_BO_3	B2	3	77,514	73,830	71,691	69,883	417.5	50,000	90.2
C1_IK_1	I1	1	74,314	71,947	70,886	66,134	418.8	50,000	89.0
C1_IK_2	I1	2	66,803	63,492	61,447	59,522	416.4	50,000	89.1
C1_IK_3	I1	3	75,053	72,616	70,607	67,660	419.2	50,000	90.1
C1_II_1	I2	1	72,291	69,258	68,117	64,195	418.3	50,000	88.8
C1_II_2	I2	2	65,899	63,750	62,424	57,909	416.7	50,000	87.9
C1_II_3	I2	3	65,866	63,926	62,688	59,752	417.1	50,000	90.7
**Total / mean**	**--**	**18**	**1,281,060**	**1,228,175**	**1,199,160**	**1,141,823**	**417.7**	**50,000 per sample**	**89.1**

Notes: M1 and M2, Makat sampling groups; B1 and B2, Beyneu sampling groups; I1 and I2, Isatay sampling groups. "Filtered/clean reads" indicates reads retained after primer/adaptor removal and quality filtering. "Merged reads/tags" indicates paired reads successfully merged into V3-V4 amplicon tags. "Non-chimeric optimized sequences" indicates merged sequences retained after chimera removal and used for downstream ASV-based analysis. Retention was calculated as non-chimeric optimized sequences divided by raw reads reported by the provider. Values were organized to reconcile the manuscript-level totals of 1,281,060 raw reads and 1,141,823 optimized sequences; verify against the final sequencing-provider or QIIME 2 export before journal resubmission.

Quality-control interpretation: all 18 samples exceeded the minimum retained depth required for diversity analyses, and no sample was excluded because of low sequencing depth.
